# Gonadotropin-releasing hormone type II (GnRH-II) agonist regulates the invasiveness of endometrial cancer cells through the GnRH-I receptor and mitogen-activated protein kinase (MAPK)-dependent activation of matrix metalloproteinase (MMP)-2

**DOI:** 10.1186/1471-2407-13-300

**Published:** 2013-06-20

**Authors:** Hsien-Ming Wu, Hsin-Shih Wang, Hong-Yuan Huang, Chyong-Huey Lai, Chyi-Long Lee, Yung-Kuei Soong, Peter CK Leung

**Affiliations:** 1 Department of Obstetrics and Gynecology, Chang Gung Memorial Hospital Linkou Medical Center, Chang Gung University School of Medicine, Taoyuan 333, Taiwan; 2 Department of Obstetrics and Gynecology, University of British Columbia, Vancouver, BC V6H3V5, Canada

**Keywords:** GnRH-II agonist, Invasion, ERK1/2, JNK, MMP-2

## Abstract

**Background:**

More than 25% of patients diagnosed with endometrial carcinoma have an invasive primary cancer accompanied by metastases. Gonadotropin-releasing hormone (GnRH) plays an important role in reproduction. In mammals, expression of GnRH-II is higher than GnRH-I in reproductive tissues. Here, we examined the effect of a GnRH-II agonist on the motility of endometrial cancer cells and its mechanism of action in endometrial cancer therapy.

**Methods:**

Immunoblotting and immunohistochemistry (IHC) were used to determine the expression of the GnRH-I receptor protein in human endometrial cancer. The activity of MMP-2 in the conditioned medium was determined by gelatin zymography. Cell motility was assessed by invasion and migration assay. GnRH-I receptor si-RNA was applied to knockdown GnRH-I receptor.

**Results:**

The GnRH-I receptor was expressed in the endometrial cancer cells. The GnRH-II agonist promoted cell motility in a dose-dependent manner. The GnRH-II agonist induced the phosphorylation of ERK1/2 and JNK, and the phosphorylation was abolished by ERK1/2 inhibitor (U0126) and the JNK inhibitor (SP600125). Cell motility promoted by GnRH-II agonist was suppressed in cells that were pretreated with U0126 and SP600125. Moreover, U0126 and SP600125 abolished the GnRH-II agonist-induced activation of MMP-2. The inhibition of MMP-2 with MMP-2 inhibitor (OA-Hy) suppressed the increase in cell motility in response to the GnRH-II agonist. Enhanced cell motility mediated by GnRH-II agonist was also suppressed by the knockdown of the endogenous GnRH-I receptor using siRNA.

**Conclusion:**

Our study indicates that GnRH-II agonist promoted cell motility of endometrial cancer cells through the GnRH-I receptor via the phosphorylation of ERK1/2 and JNK, and the subsequent, MAPK-dependent activation of MMP-2. Our findings represent a new concept regarding the mechanism of GnRH-II-induced cell motility in endometrial cancer cells and suggest the possibility of exploring GnRH-II as a potential therapeutic target for the treatment of human endometrial cancer.

## Background

Endometrial cancer is one of the most common gynecological cancers in the world and accounts for approximately 50,000 deaths worldwide each year [1]. Patients with tumor confined to the uterus are treated with surgery and radiotherapy [2-4]. However, more than 25% of patients diagnosed with endometrial carcinoma have an invasive primary cancer accompanied by metastases. Despite treatment with aggressive chemotherapeutic regimens, these patients have a 5-year survival rate of less than 20% [1]. In fact, metastasis represents the main cause of death for patients with endometrial cancer, and the battle against this cancer would greatly benefit from the identification of factors involved in the metastatic process. Certain cases of endometrial cancer with a particular morphology, adverse histopathological features or advanced stage are characterized by aggressive behavior and poor prognosis [5]. The molecular pathogenesis of endometrial cancer remains poorly understood, resulting in a limited cure rate in the treatment of advanced cases. Thus, new therapeutic approaches are needed for advanced or relapsed disease. The hypothalamic peptide GnRH plays an important role in the maintenance of intrauterine tissues and the development of endometrial cancer [6-9]. In mammals, GnRH-II is more widely present in peripheral tissues than GnRH-I, which suggests that GnRH-II may have additional functions. GnRH-II has been shown to have direct antiproliferative effects in the growth of endometrial cancer cells [10]. These findings raise the possibility that GnRH-II could directly regulate the tumor progression of endometrial cancer cells. The role of GnRH-II in endometrial cancer cell invasion is not known, and the mechanism by which GnRH-II regulates the invasiveness of endometrial tumors has also not been established. The MAPKs are considered to be important components of GnRH-induced signaling pathways in various cell types [10-12]. We have previously demonstrated that the anti-proliferative effect of GnRH-II is mediated by the MAPKs signalings [10,13]. Different mechanisms have been suggested for MAPK activation through GPCRs [14,15]. MMPs are largely implicated in promoting angiogenesis and tumor metastasis [16,17]. Some evidence indicates an expanded role for GnRH in certain aspects of gynecologic tumor progression, such as metastasis, via the activation of MMPs and the subsequent increase in cell migration and invasion [18]. In the present study, we examined the effect of a GnRH-II agonist on the motility of endometrial cancer cells and the mechanisms of the action involved. Our results suggest the possibility of exploring GnRH-II as a potential therapeutic target for the treatment of human endometrial cancer.

## Results

### GnRH-II stimulates migration and invasion of endometrial cancer cells

**Figure 1:**
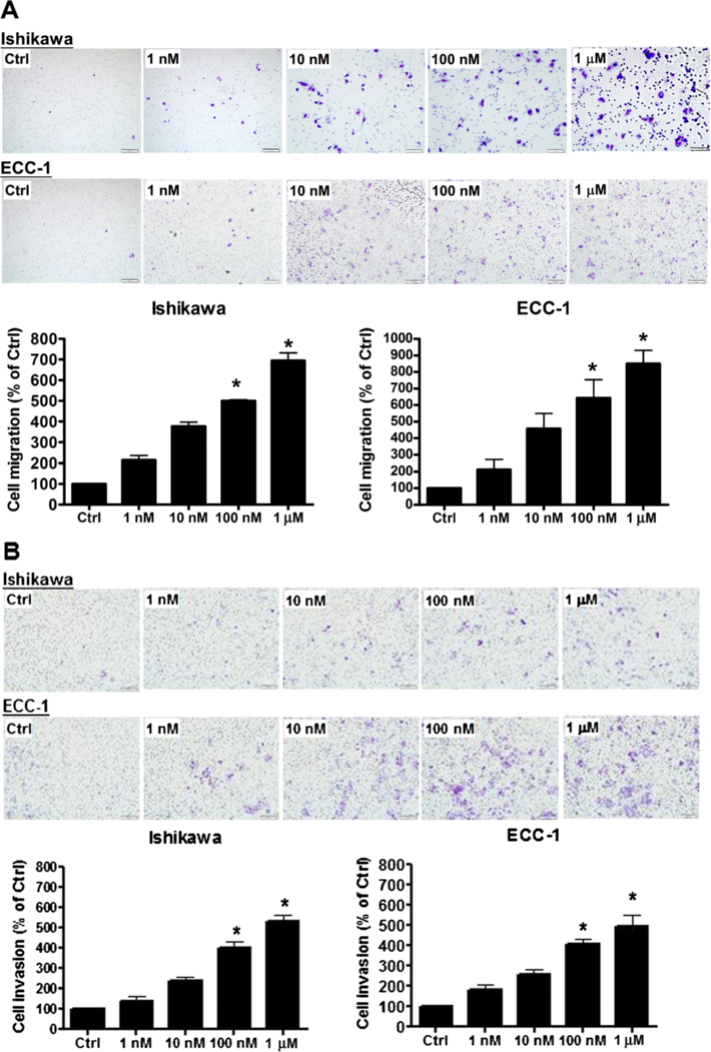
**GnRH-II stimulates endometrial cancer cell migration and invasion. (A)** Using the Transwell migration assay, endometrial cancer cells were seeded. The GnRH-II agonist stimulated the migration of endometrial cancer cells through the uncoated porous filter in a dose-dependent manner at concentrations of 1 nM to 1 μM with a maximal effect at 1 μM. **(B)** Endometrial cancer cells were seeded onto a Matrigel-precoated filter in the Transwell chambers in the presence or absence of increasing concentrations of GnRH-II agonist (1 nM to 1 μM, as indicated). After 24 (migration) and 48 (invasion) hours of incubation, cells in the upper side of the filter were removed and the migrated or invaded cells were fixed, stained, and counted. Left, representative pictures. Columns, the mean number of migrated or invaded cells of five fields of triplicate wells from three independent experiments; bars, SD; *p<0.05, versus control.

In cancer invasion and metastasis, an imbalanced regulation of cell motility and proteolysis appears to be a critical event [19]. To study whether the expression of the GnRH-I receptor is associated with the metastasis of endometrial cancer cells, the effect of GnRH-II on cell migration and invasion was examined. Ishikawa and ECC-1 endometrial cancer cells, which express functional GnRH-I receptors [10], were treated with a GnRH-II agonist. The ability of the cells to migrate was assessed using a Transwell migration assay. The GnRH-II agonist stimulated the migration of endometrial cancer cells through the uncoated porous filter in a dose-dependent manner at concentrations of 1 nM to 1 μM with a maximal effect at 1 μM (Figure 1A). We also assessed the invasion of the cells in vitro in response to the GnRH-II agonist stimulus using Transwells with filters coated with Matrigel. Our results indicated that the GnRH-II agonist induced endometrial cancer cell invasion in a dose-dependent manner at concentrations of 1 nM to 1 μM with a maximal effect at 1 μM (Figure 1B).

### Expression of the GnRH-I receptor (GnRH-IR) in endometrial cancer

**Figure 2:**
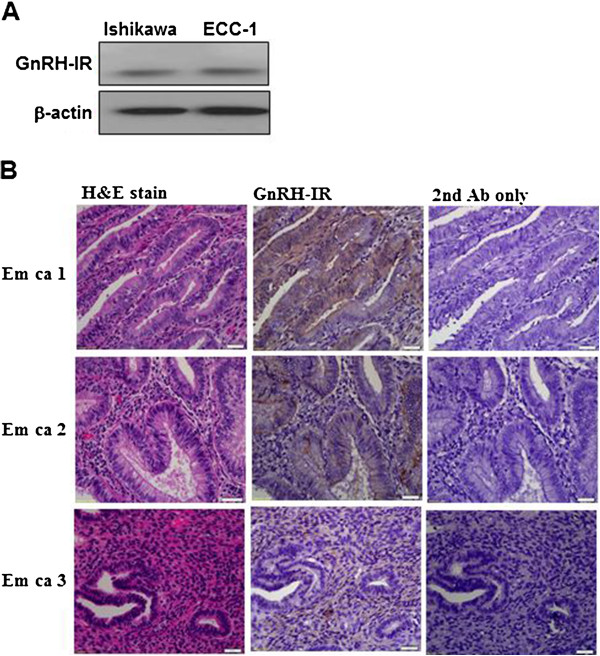
**Immunoblot and immunohistochemical analysis of GnRH-I receptor (GnRH-IR) protein expression in endometrial cancer. (A)** GnRH-I receptor levels in Ishikawa and ECC-1 cells were monitored by immunoblot assays. **(B)** GnRH-I receptor was stained brown in the second of three columns depicting human endometrial cancer tissue sections. Sections were counterstained with hematoxylin to show the nuclei in column 1 of three columns depicting human endometrial cancer tissue sections. Sections were stained without the GnRH-I receptor antibody as a negative control in the third of three columns depicting human endometrial cancer tissue sections. Micrographs were taken with a 40Χ objective lens. Scale bars represent 20 μm.

To examine the expression of the GnRH-I receptor, Ishikawa and ECC-1 endometrial cancer cells were lysed, and the expression of GnRH-I receptor was examined by immunoblot analysis. As shown in Figure 2A, the GnRH-I receptor was detected in Ishikawa and ECC-1 endometrial cancer cells. Using immunohistochemical analysis, we confirmed that the GnRH-I receptor was expressed in the human endometrial cancer tissue samples (Figure 2B).

### The GnRH-II-induced cell migration and invasion is mediated by GnRH-I receptors in endometrial cancer cells

**Figure 3:**
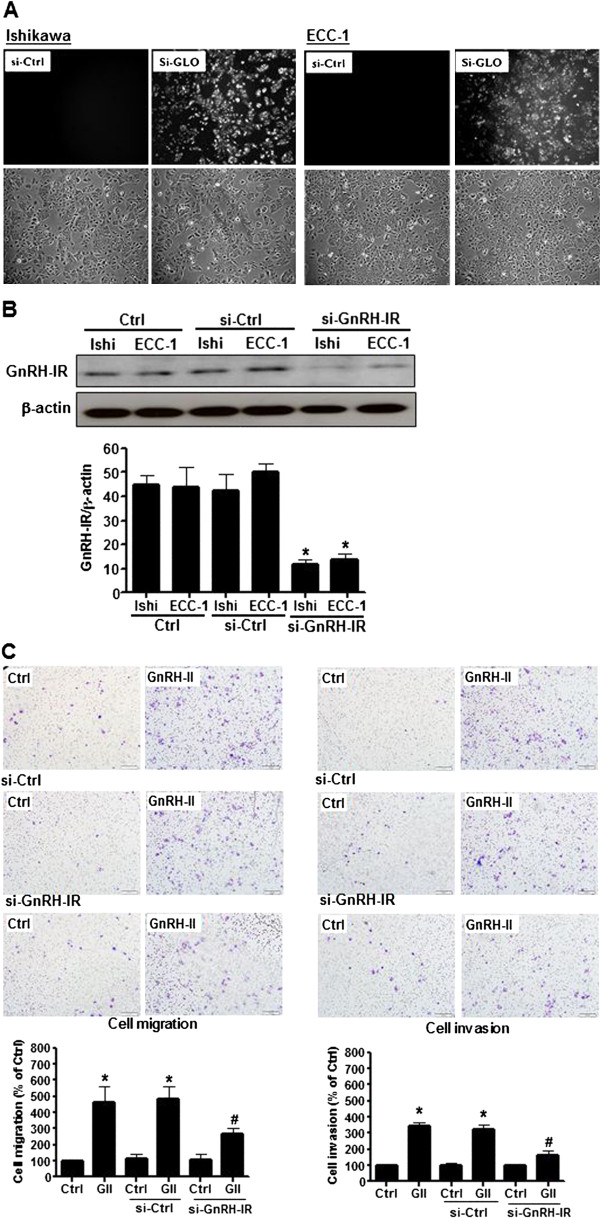
**Effects of human GnRH-I receptor siRNA (si-GnRH-IR) transfection on endometrial cancer cells. (A)** The transfection efficiency of siRNA in endometrial cancer cells. **(B)** GnRH-I receptor levels were monitored by immunoblot assays. The endometrial cancer cells were transfected with human si-GnRH-IR or scrambled siRNA (si-Ctrl) for one day with Lipofectamine RNAiMAX. **(C)** The effects of si-GnRH-IR transfection on the GnRH-II-induced cell migration. The cells were transfected with si-GnRH-IR and treated with GnRH-II (1 μM) for 24 h. The cell motility was assessed with the migration assay. The results are expressed as the mean ± SEM of three independent experiments. (*p<0.05, versus control; #p<0.05, versus GnRH-II). The effects of si-GnRH-IR transfection on GnRH-II-induced cell invasion. The cells were transfected with si-GnRH-IR and treated with GnRH-II (1 μM) for 48 h. The cell motility was assessed with the invasion assay. The results are expressed as the mean ± SEM of three independent experiments. (*p<0.05, versus control; #p<0.05, versus GnRH- II).

It is assumed that both GnRH-I and GnRH-II exert their biological effects by binding to a common GnRH-I receptor [20]. To investigate whether the effects of GnRH-II on cell migration and invasion were mediated by the GnRH-I receptor, Ishikawa and ECC-1 endometrial cancer cells were transfected with a GnRH-I receptor siRNA to knockdown the endogenous GnRH-I receptor expression. The trnasfection efficiency of siRNA in both Ishikawa and ECC-1 was examined by using fluorescence-labeling siRNA, si-GLO. As shown in Figure 3A, both cells were almost transfected after 24 hours si-GLO transfection. Treatment with 50 nM GnRH-I receptor siRNA down-regulated GnRH-I receptor expression in Ishikawa and ECC-1 endometrial cancer cells (Figure 3B). Moreover, knockdown of the endogenous GnRH-I receptor significantly abolished the GnRH-II-mediated cell migration (Figure 3C) and abolished the GnRH-II-promoted cell nvasion. Taken together, these results indicate that the GnRH-II-induced cell migration and invasion in endometrial cancer cells are mediated by GnRH-I receptors.

### GnRH-II-induced cell migration and invasion are mediated by ERK1/2 and JNK signaling in endometrial cancer cells

**Figure 4:**
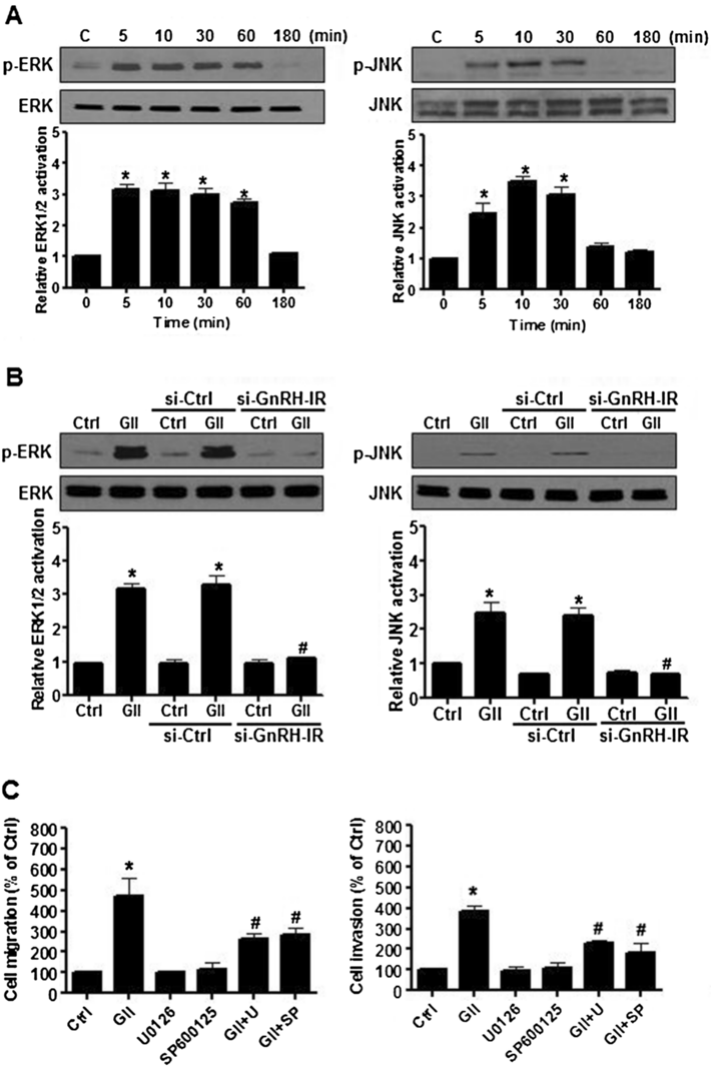
**The effects of ERK1/2 and JNK signaling in endometrial cancer cells. (A)** The effects of GnRH-II on ERK1/2 and JNK signaling activation. The cells were treated with GnRH-II (1 μM) at different time points. The phosphorylated ERK1/2 (p-ERK1/2), and phosphorylated JNK (p-JNK) levels were analyzed by immunoblot analysis, which indicated increases in the levels of p-ERK1/2 and p-JNK following 5 min of stimulation. **(B)** The effects of human si-GnRH-IR transfection on the GnRH-II-induced activation of ERK1/2 and JNK. The activation of ERK1/2 and JNK induced by GnRH-II (GII) was investigated after si-GnRH-IR transfection and showed significant decreases in the levels of p-ERK1/2 and p-JNK. **(C)** The effects of U0126 and SP600125 pretreatment on GnRH-II-induced ERK1/2 and JNK activation. Cells were pretreated individually with 1 μM U0126 or 1 μM SP600125 for 30 min followed by stimulation with 1 μM GnRH-II for 10 min. The control culture was treated with DMSO as a vehicle control. Pretreatment with 1 μM U0126 and 1 μM SP600125 individually attenuated the effects of GnRH-II on the induction of cell migration and invasion. (Columns, mean from three independent experiments in three different passages of the cell line; bars, SE., *p<0.05, versus control; #p<0.05, versus GnRH-II).

To investigate the molecular mechanism of GnRH-II-induced cell migration and invasion in endometrial cancer cells, the activation of ERK1/2 and JNK signaling were examined with immunoblot analysis. As shown in Figure 4A, GnRH-II activated ERK1/2 and JNK signaling in a time-dependent manner. The effects of GnRH-II on ERK1/2 and JNK signaling activation were abolished by transfecting the cells with GnRH-IR siRNA but not with control siRNA (Figure 4B). To further evaluate the roles of ERK1/2 and JNK signaling in GnRH-II-induced cell migration and invasion, endometrial cancer cells were treated with U0126 and SP600125 along with GnRH-II. As shown in Figure 4C, pretreatment of the cells with U0126 or SP600125 abolished the GnRH-II-stimulated cell migration and invasion. These results suggest that GnRH-II induced the cell migration and invasion of endometrial cancer cells through the GnRH-I receptor and the activation of the ERK1/2 and JNK signaling pathways.

### Effects of GnRH-II-induced MMP-2 expression on the cell migration and invasion of endometrial cancer cells

**Figure 5:**
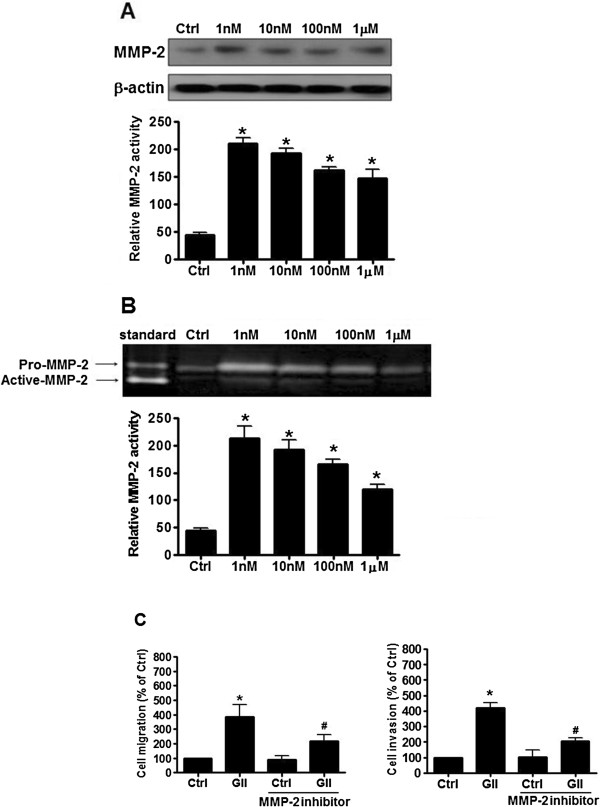
**The effects of GnRH-II on MMP-2 expression in endometrial cancer cells. (A)** GnRH-II increased MMP-2 protein expression in endometrial cancer cells. **(B)** Conditioned medium from the treated cells was also collected and analyzed for MMP activity with gelatin zymography. Arrows, gelatinase activities corresponding to pro-MMP-2, and active-MMP-2. Lane 1, a human MMP-2 standard was used as a positive control. **(C)** The cells were pretreated with MMP-2 inhibitor. Pretreated cells were collected for the migration assay through uncoated filter (left) and the invasion assay through Matrigel (right) in the presence or absence of GnRH-II. The results are expressed as the mean ± SEM of three independent experiments. (*p<0.05, versus control; #p<0.05, versus GnRH-II).

MMP-2 is largely implicated in promoting angiogenesis and tumor metastasis. To determine whether MMP-2 is involved in GnRH-II-induced cell migration and invasion of endometrial cancer cells, the cells were treated with GnRH-II, and the expression of MMP-2 was detected by immunoblot analysis. As shown in Figure 5A, treatment with 1 nM to 1 μM GnRH-II obviously induced MMP-2 expression. Furthermore, MMP-2 enzymatic activity was measured by gelatin zymography using conditioned medium from endometrial cancer cells. The gelatin zymography indicated stronger lytic zones at the molecular masses corresponding to the pro- and active-forms of MMP-2 (the 72-kDa and 66-kDa forms, respectively) in the conditioned medium from cells treated with 1 nM to 1 μM GnRH-II compared with that from untreated cells (Figure 5B). A more important observation was that the GnRH-II-induced cell migration and invasion were abolished in cells pretreated with the MMP-2 inhibitor, indicating that MMP-2 was critical for the effects of GnRH-II on the cell migration and invasion of endometrial cancer cells (Figure 5C).

## Discussion

The GnRH pathway is important in the hypothalamus-pituitary-gonadal axis of reproduction [21]. Previous studies have demonstrated the direct effects of GnRH analogs in human endometrial cancer cells [22,23]. Furthermore, it has been demonstrated that GnRH-II has more potent effects than GnRH-I in extra-pituitary tissues, such as endometrial tumors, suggesting that GnRH-II could be considered as a possible therapeutic target for endometrial cancers [22]. Metastasis represents the main cause of death for patients with endometrial cancer, and the battle against this cancer would benefit greatly from the identification of factors involved in the metastatic process. However, the underlying molecular mechanisms utilized by GnRH-II to regulate the cell migration and invasion of endometrial cancer are not well known. The GnRH-I receptor is a member of the GPCR family. GPCRs are characterized by the presence of seven transmembrane domains and transfer their signals through multiple G protein subunits, often stimulating multiple signaling pathways [24]. Direct evidence showing the presence of a full-length, functional GnRH-II receptor mRNA in human tissues is insufficient, and the issue of whether the GnRH-I receptor mediates the effects of both GnRH-I and GnRH-II remains unresolved. In this study, we report for the first time that GnRH-II may contribute to the migration and invasion of endometrial cancer cells by inducing the expression of MAPK-mediated MMP-2 through the GnRH-I receptor, providing an insight into the prospect of developing targeted therapy for endometrial cancer.

In our previous study [10], the expression of GnRH-II and its effects on cell growth were demonstrated in endometrial cancer. In the present study, the treatment of Ishikawa and ECC-1 endometrial cancer cells with GnRH-II resulted in significant effects on cell migration and invasion. These findings suggest that GnRH-II directly induces the cell migration and invasion of endometrial cancer cells and provide in vitro confirmation that GnRH-II induces cell motility in endometrial cancer. These findings confirmed the previous studies [22,23,25-29] suggesting that GnRH-II may mediates the cell motility and anti-proliferation in gynecologic cancer cell lines. Therefore, differences in levels of GnRH-I receptor, GnRH-II receptor and signaling differentially affect the apoptotic and motile machinery within cell lines and contribute to the cell type–specific effects of GnRH analogues on cell growth and motility.

In this study, GnRH-I receptor siRNA was used to selectively knock down the protein expression of GnRH-I receptors in Ishikawa and ECC-1 endometrial cancer cells. Targeting GnRH-I receptors with siRNA abolished the GnRH-II-induced cell migration and invasion of endometrial cancer cells, indicating that the effects of GnRH-II on endometrial cancer cells is dependent upon GnRH-I receptors. This finding confirmed previous studies [10,30-32] that suggested that the GnRH-I receptor may be a common receptor that mediates the effects of both GnRH-I and GnRH-II in gynecological cancer cells.

In pituitary gonadotrope cells, MAPKs are considered to be essential in GnRH-induced signaling pathways [12,33]. MAPKs contribute to signaling pathways that mediate cellular responses to different extracellular stimuli and thereby determine the cell’s behavior. In the present study, we observed that GnRH-II (1 μM) resulted in the phosphorylation of ERK1/2 and JNK in Ishikawa endometrial cancer cells, which is compatible with a previous study performed in COS-7 cells [34]. Moreover, the activation of ERK1/2 and JNK was markedly attenuated by the specific inhibitors U0126 and SP600125 in Ishikawa endometrial cancer cells. Treatment with U0126 and SP600125 also attenuated the GnRH-II-induced cell migration and invasion, further indicating that the GnRH-II-induced activation of ERK1/2 and JNK may have an important role in the regulation of cell motility in Ishikawa endometrial cancer cells. The present results indicate that the ERK1/2 and JNK pathways might play an important role in mediating the motility effects of GnRH-II in Ishikawa endometrial cancer cells. Therefore, attempts to manipulate the ERK1/2 and JNK signaling that mediates the regulation of cell migration and invasion may be an approach to explore the effects of GnRH-II in endometrial cancer.

Cancer cell metastasis is a complex process that involves proteolysis, increased cell motility, and decreased cell adhesion. MMP-2 has been suggested to play a critical role in cancer metastasis, and the up-regulation of MMP-2 is associated with increased invasion and a poor prognosis in cancer [35-38]. In addition to their enzymatic activities, MMPs can also promote cancer cell migration by influencing cytoskeletal organization through their association with different families of adhesion receptors [39]. In the present study, we demonstrated that GnRH-II promotes the cell migration and invasion of endometrial cancer cells through the increased expression and proteolytic activity of MMP-2, which specifically degrades the basement membrane. Pretreatment with U0126 and SP600125 abolished the protein expression of MMP-2 induced by GnRH-II, suggesting that the ERK1/2 and JNK signaling pathways may play an important role in regulating MMP-2 expression. Taken together with the previous results, the cell migration and invasion in endometrial cancer is regulated by the activation of the ERK1/2 and JNK signaling pathways by GnRH-II and is accompanied by the induction of MMP-2. This is one of the novel findings in the present study. In aggregate, our data demonstrate that MMP-2 is closely associated with the pathways of the MAPKs involved in the GnRH-II-induced cell migration and invasion of endometrial cancer cells. Targeting MMP-2 with an MMP-2 inhibitor blocked the GnRH-II-induced cell migration and invasion, indicating that the effects of GnRH-II in endometrial cancer cells are strongly correlated with MMP-2 expression.

## Conclusions

In conclusion, our findings suggest that the potential role of GnRH-II in promoting the cell migration and invasion of endometrial cancer is through the binding of GnRH-I receptors, the activation of the ERK1/2 and JNK pathways, and the subsequent induction of the metastasis-related proteinase MMP-2 activity. This information provides a mechanistic rationale for the observed GnRH-I receptor expression in endometrial cancer. Our findings provide a new insight regarding the mechanism of GnRH-II-induced cell motility in endometrial cancer and suggest the possibility of exploring GnRH-II as a potential therapeutic molecular target for the treatment of human endometrial cancer.

## Methods

### Cell lines and cell culture

The human endometrial cancer cell lines Ishikawa and ECC-1 were utilized in this study. The human endometrial cancer cell line Ishikawa is a well-differentiated endometrial adenocarcinoma cell line [40]. The ECC-1 cell line, derived from a well-differentiated adenocarcinoma of the endometrium [41], was obtained from the American Type Culture Collection (US). The cells were cultured in Dulbecco’s minimum essential medium (DMEM) with 10% fetal bovine serum (FBS; Hyclone Laboratories Inc., Logan, UT), 100 U/ml penicillin, and 100 μg/ml streptomycin and incubated at 37°C in a humidified incubator with 5% CO_2_. The cells were grown to 80% confluence and transferred to serum-free medium for 24 h prior to the treatment with the GnRH-II agonist.

### Reagents

The GnRH-II agonist (D-Arg6, AzaGly10-GnRH II), a synthetic decapeptide, was purchased from Bachem (San Carlos, CA). The MAPK/extracellular signal-regulated protein kinase (ERK) kinase (MEK) inhibitor U0126, the JNK inhibitor SP600125, and the MMP-2 inhibitor OA-Hy were purchased from Calbiochem (San Diego, CA).

### Immunoblot analysis

The cells were lysed in buffer containing 20 mM Tris, pH 7.4, 2 mM EGTA, 2 mM Na_2_VO_3_, 2 mM Na_4_P_2_O_7_, 2% Triton X-100, 2% SDS, 1 μM aprotinin, 1 μM leupeptin and 1 mM PMSF. The protein concentration was determined with a protein assay kit using BSA standards according to the manufacturer's instructions (Bio-Rad Laboratories, Hercules, CA). Equal amounts of cell lysate were separated by SDS polyacrylamide gel electrophoresis (PAGE) and transferred to a nitrocellulose membrane (Hybond-C, Amersham Pharmacia Biotech Inc., Oakville, ON). Following blocking with Tris-buffered saline (TBS) containing 5% non-fat dry milk for 1 h, the membranes were incubated overnight at 4°C with anti-GnRH-I receptor (Neomarker, Fremont, CA), anti-phospho-ERK1/2 (Cell signaling), anti-ERK1/2 (Cell signaling), anti-phospho-JNK (Cell signaling), anti-JNK (Cell signaling), or anti-MMP-2 (Calbiochem, San Diego, CA) antibody followed by incubation with HRP-conjugated secondary antibody. The immunoreactive bands were detected with an enhanced chemiluminescence (ECL) kit. The membrane was then stripped with stripping buffer (62.5 mM Tris, 10 mM DTT, and 2% SDS, pH 6.7) at 50°C for 30 min and re-probed with anti-β-actin antibody (Santa Cruz) as a loading control.

### Immunohistochemistry (IHC)

To determine the expression of the GnRH-I receptor protein in human endometrial cancer, IHC was performed on sections of human endometrial cancer tissue using previously reported procedures [42]. The involvement of human subjects in this study was approved by the Institutional Review Board of Chang Gung Memorial Hospital (CGMH-IRB numbers 101-2187B and 100-3879C). Four-micrometer-thick formalin-fixed, paraffin-embedded (FFPE) tissue sections were deparaffinized in xylene and rehydrated with a graded series of ethanol solutions. The sections were then stained with an anti-human GnRH-I receptor polyclonal antibody (Neomarker; 1:100) using an automated IHC stainer with the Ventana Basic DAB Detection kit (Tucson, AZ) according to the manufacturer’s protocol. Counterstaining was performed with hematoxylin. Sections were stained without the GnRH-I receptor antibody as a negative control in the third of three columns depicting the human endometrial cancer tissue sections.

### Small interfering RNA transfection

siGENOME ON-TARGETplus SMARTpool human GnRH-I receptor siRNA and siCONTROL NON-TARGETINGpool siRNA were purchased from Dharmacon. The cells were transfected with siRNA (100 nM) using Lipofectamine RNAiMAX. After a 24 h transfection, the medium was removed and changed to fresh serum-free medium. To examine the siRNA transfection, cells were transfected with 100 nM si-GLO (Dharmacon) for 24 hr. The transfection efficiency was examined by fluorescent microscopy.

### Invasion and migration assays

Migration and invasion assays were performed in Boyden chambers with minor modifications. Cell culture inserts (24-well, pore size 8 μm; BD Biosciences, Mississauga, ON) were seeded with 1x105 cells in 250 μL of medium with 0.1% FBS. Un-coated inserts were used for migration assays whereas inserts pre-coated with growth factor reduced Matrigel (40 μl, 1 mg/ml; BD Biosciences) were used for invasion assays. Medium with 10% FBS (750 μl) was added to the lower chamber and served as a chemotactic agent. After 24 hr (migration) or 48 hr (invasion) incubation, non-migrating/invading cells were wiped from the upper side of the membrane and cells on the lower side were fixed in cold methanol (−20°C) and air dried. The cells that had not penetrated the filter were removed by wiping, and the cells that had invaded the lower surface of the filter were fixed with ice-cold methanol and stained with 0.5% crystal violet.

### Gelatin zymography

The activity of MMP-2 in the conditioned medium was determined by gelatin zymography. The media were collected and clarified by centrifugation to remove cells and debris. The samples were loaded under non-reducing conditions onto SDS-polyacrylamide gel polymerized with 1 mg/mL gelatin. Following electrophoresis, the gels were washed with 2.5% Triton X-100 to remove SDS and then incubated in a developing buffer overnight at 37°C. The gels were stained with 0.25% Coomassie Brilliant Blue R-250 and destained in the same solution without dye. The gelatinase activity was visualized as clear bands against the blue-stained gelatin background. The molecular sizes were determined from mobility using gelatin zymography standards.

### Statistical analysis

The results are shown as the means ± SEM. Statistical evaluation was conducted with the *t*-test for paired data. Multiple comparisons were first analyzed by one-way ANOVA, followed by Tukey’s multiple comparison test. A significant difference was defined as p<0.05.

## Competing interests

The authors declare that they have no competing interests.

## Authors’ contributions

HMW, HSW, and PCKL performed the experiments, interpreted the results and prepared the manuscript. HMW, HSW, HYH, CHL, CLL, YKS, and PCKL contributed to the scientific discussion and the manuscript editing. HSW and PCKL supervised in the design of the study and finalized the manuscript. All authors read and approved the final manuscript.

## Pre-publication history

The pre-publication history for this paper can be accessed here:

http://www.biomedcentral.com/1471-2407/13/300/prepub
